# TARPγ2-Derived Peptide Enhances Early-Phase Long-Term Potentiation and Impairs Memory Retention in Male Rats

**DOI:** 10.3390/brainsci15080881

**Published:** 2025-08-18

**Authors:** Dominik Mátyás, Vanda Tukacs, Vilmos Tóth, Péter Baracskay, Stefánia Krisztina Pap, Pál Stráner, Trần Minh Hiền, Éva Hunyadi-Gulyás, Zsuzsanna Darula, András Perczel, Katalin Adrienna Kékesi, Gábor Juhász

**Affiliations:** 1 Laboratory of Proteomics, Institute of Biology, ELTE Eötvös Loránd University, Pázmány Péter Sétány 1/C, H-1117 Budapest, Hungarykakekesi@ttk.elte.hu (K.A.K.); gjuhasz100@gmail.com (G.J.); 2 Department of Physiology and Neurobiology, Institute of Biology, ELTE Eötvös Loránd University, Pázmány Péter Sétány 1/C, H-1117 Budapest, Hungary; 3 InnoScience Hungary Ltd., Bátori út 9, H-3143 Mátranovák, Hungary; 4 ELTE NAP Neuroimmunology Research Group, Department of Biochemistry, Institute of Biology, ELTE Eötvös Loránd University, H-1117 Budapest, Hungary; 5 Laboratory of Structural Chemistry and Biology, Institute of Chemistry, ELTE Eötvös Loránd University, Pázmány Péter Sétány 1/A, H-1117 Budapest, Hungary; 6 HUN-REN–ELTE Protein Modeling Research Group, ELTE Eötvös Loránd University, Pázmány Péter Sétány 1/A, H-1117 Budapest, Hungary; 7 Core Facility Proteomics Research Group, HUN-REN Biological Research Centre, Temesvári Körút 62, H-6726 Szeged, Hungary; 8 Single Cell Omics Advanced Core Facility, Hungarian Centre of Excellence for Molecular Medicine, Budapesti út 9, H-6728 Szeged, Hungary

**Keywords:** synaptic plasticity, long-term potentiation, spatial memory, protein-protein interaction, TARPγ2, Arc

## Abstract

**Background/Objectives**: Disruption of AMPAR trafficking at excitatory synapses contributes to impaired synaptic plasticity and memory formation in several neurological and psychiatric disorders. Arc, an immediate early gene product, has been shown to interact with the AMPAR auxiliary subunit TARPγ2, affecting receptor mobility and synaptic stabilization. **Methods**: To investigate the in vivo functional effects and protein interactions of the Arc-TARPγ2 interfering peptide RIPSYR, we performed in vivo electrophysiology and spatial memory assessments in male rats. as well as proteomic analyses of peptide-protein interactions in synaptosome lysates. We then used in silico docking to evaluate candidate binding partners. **Results**: In the present study, in vivo electrophysiological measurements revealed that RIPSYR administration altered early-phase long-term potentiation at CA3 synapses of male rats. Subsequent behavioral testing that assessed spatial memory performance revealed depleted memory retrieval after 24 h, indicating that the peptide has a systemic effect on experience-dependent plasticity. Then, we examined the molecular interactome of RIPSYR using magnetic bead-based immunoprecipitation and subsequent LC-MS identification on synaptosome lysates, and identified additional candidate binding partners, suggesting that the peptide may have broader modulatory effects. RIPSYR binding to the other putative binding partners are investigated by in silico methods. **Conclusion**: Our results raise the question of how the molecular interactions of RIPSYR contribute to its sum effects on electrophysiology and behavior.

## 1. Introduction

The molecular mechanism of memory is one of the fundamental problems in neuroscience. Memory is a multi-step process including the encoding, consolidation, and the long-term storage of the memory trace. Impairments of these processes are a hallmark of several neuropsychiatric and neurodegenerative diseases [1,2]. Despite the increasing burden of these conditions and the vast amounts of data that are collected about the molecular mechanisms of memory inscription and consolidation, the transfer of the initial memory trace to a sustained and retrievable long-term trace remains limited. Rapidly developing knowledge about protein–protein interactions and development of protein science uncovered details of dynamic modifications in synaptic proteins in in vitro studies; however, several of these are not confirmed in vivo. This is particularly true for interaction-based putative mechanisms of receptor mobility, internalization, and externalization.

The most widely studied model of stimulus-induced synaptic molecular rearrangement is long-term potentiation (LTP) and long-term depression (LTD) [3,4]. It is known that LTP and LTD, are directly coupled to AMPAR (α-amino-3-hydroxy-5-methyl-4-isoxazolepropionic acid receptor) number, permeability and intra-synaptic distribution in synaptic protein nanoclusters [5,6]. Synaptic strength is tuned dynamically by increasing or decreasing the number of AMPARs and modulating their spatial distribution in the postsynaptic membrane [7]. A large amount of data supports the role of AMPAR mobility in synaptic plasticity, which is regulated by synaptic activity [7,8]. Stimulus-induced synaptic modulation is based on AMPAR mobility while the anchoring of AMPARs at their new location is the basis of sustaining the change in transmission.

AMPAR mobility and anchoring into the synaptic scaffold are based on protein complexes as revealed by in vitro [9,10] and in vivo studies [11]. AMPAR mobility is regulated by auxiliary proteins, which are responsible for binding AMPAR to the synaptic scaffold [12,13]. Members of the transmembrane AMPAR regulatory protein (TARP) family are regulatory subunits that bind AMPARs [14,15]. TARPγ-2 binds postsynaptic density protein 95 (PSD95) simultaneously through its PDZ-binding C-terminal domain [16,17], which temporarily anchors the receptor to the active surface of the post-synaptic membrane [18,19]. The activity-regulated cytoskeleton associated protein (Arc) binds dephosphorylated TARPγ2 proteins at the TARPγ2 C-terminal (225-RIPSYR-230) region evidenced by an in vitro fluorescence polarization assay study [20], and it detaches the TARPγ2-AMPAR protein complex from PSD95 via phase separation in an in vitro reconstituted PSD condensate model [21]. Among the TARP isoforms, the conserved binding motif is found in γ2, γ4, and γ8, with both TARPγ2 and TARPγ8 shown to bind Arc in vitro [21]. However, distinct intracellular domains of TARPγ2 and TARPγ8 differentially regulate AMPAR trafficking and gating [15]. We focus on TARPγ2 because its interaction with Arc has been most extensively characterized in hippocampal neurons. Binding of Arc to the TARP-AMPAR complex results in greater AMPAR dispersion at the postsynaptic density [21] by inhibiting AMPAR anchoring. Through this process, Arc can downscale synaptic strength and protect synapses against saturation while preserving their plasticity [22]. Thus, Arc has a pivotal role in synaptic plasticity and learning by maintaining homeostatic synaptic scaling [23,24]. The molecular mechanism described above was discovered in biochemical and cell culture studies; however, it is still unclear how protein–protein interactions that regulate AMPAR mobility are directly linked to physiological plasticity in the LTP model, as well as their contribution to behavioral learning. Hence, inhibiting the Arc-TARPγ2 binding might be a valuable tool to study the physiological and behavioral outcomes of AMPAR anchoring regulation. We aimed to uncover how this binding inhibitor peptide, designed to act on the AMPAR anchoring, affects LTP and contributes to behavioral learning outcomes in vivo.

To create a binding inhibitor peptide of Arc-TARPγ2 dimerization, the protein binding epitope of Arc and the binding epitope of TARPγ2 must be considered. Arc is an immediate early gene product, which is expressed upon neuronal activity, and encodes a “domesticated” retroviral protein. Its sequence resembles the HIV Gag protein, and as a Gag homolog protein, it is capable of capsid formation [25]. Arc is also able to bind several proteins including AMPAR and N-methyl-D-aspartate receptor (NMDAR) subunits and proteins belonging to TARPγ family [20,26]. Arc has a consensus protein binding motif: X-P-Z-(Y/F) (where X = [M, I, L, or V] and Z = [S, T, R, K, M, I, L, or V) [20,26]. Nielsen et al. (2019) showed that the GluN2APTY peptide (LSNLPTYSGHFTMR)—derived from an NMDAR subunit—suppressed oligomerization of the Arc capsid domain [26]. A short peptide epitope of TARPγ2, RIPSYR, binds Arc at the same binding site as GluN2APTY [26], and molecular dynamic simulation showed that the N-terminal domain of Arc N-lobe was more flexible without RIPSYRYR—a longer version of TARPγ2-derived peptide [27]. Thus, this molecular tool, the RIPSYR peptide, aims to prevent Arc-TARPγ2 binding while preserving TARPγ2-AMPAR binding. Short, charged peptides are taken up actively by peptide transporters [28]. All data on RIPSYR listed above was derived from in vitro studies leaving the question open of how RIPSYR can influence physiological plasticity and learning in vivo.

In the present study, we investigated the effect of RIPSYR on synaptic plasticity, via recording hippocampal LTP, and on spatial memory using a cheeseboard maze task in rats. We revealed that RIPSYR treatment induced changes in the early phase of LTP. Next, we studied the effects of RIPSYR on spatial memory and found that RIPSYR had a negative impact on spatial memory retention as tested by retrieval of goal-associated spatial memories after 24 h. Because RIPSYR is a short peptide, the likelihood of off-target interactions in vivo with both extracellular and intracellular proteins should be considered. To investigate possible off-target binding partners of RIPSYR, we performed a pulldown assay on hippocampus synaptosome lysate followed by mass spectrometry analysis. By applying in silico modelling, we estimated the enthalpy changes upon RIPSYR binding to AMPA-selective glutamate receptor 2 (GluA2) and GABA receptor subunit alpha 1 (Gabra1) and 2 (Gabra2).

Collectively, our data suggest that RIPSYR has consistent physiological and behavioral effects. However, based on the results of our pulldown assays, the enrichment of multiple synaptic proteins like Basp1, Cdc42 Gabra1/2 and GluA2, other off-target interactions of RIPSYR may contribute to its in vivo effects.

## 2. Materials and Methods

### 2.1. Peptide Synthesis

Peptides RIPSYR and NVILQIG were synthesized by a flow peptide apparatus (HPPS-4000, METALON Ltd., Budapest, Hungary). To validate RIPSYR binding to the protein-binding domain of Arc, we used the peptide NVILQIG derived from Arc (26-NILQIG-32) designed to target the oligomerization domain involved in capsid formation. The flow system and procedure were described earlier in detail [29]. Briefly, the system utilized the Fmoc/tBu strategy. A PEEK chromatography column acted as the fixed bed reactor with Fmoc-Rink amide TentaGel as the resin (0.23 mmol/g) (Iris Biotech GmbH, Marktredwitz, Germany) and dimethylformamide as the solvent (Novochem, Hungary). Peptides were cleaved off the resin by a cleavage solution (95 % trifluoroacetate (TFA), 2.5% triisopropylsilane, 2.5% H_2_O), with rigorous mixing at room temperature for 3 h. Crude peptides were then purified by RP-HPLC on a C18 column (Phenomenex, Torrance, CA, USA) with a water/acetonitrile gradient (eluent A: 0.1% TFA in water, eluent B: 0.08% TFA, 80% acetonitrile in water). Peaked fractions were lyophilized, then the peptide mass was verified by an amaZon SL mass spectrometer (Bruker, Billerica, MA, USA) equipped with an electrospray ionization source. Pure fractions were stored at −4 °C.

### 2.2. Animals

Male Wistar rats (*n* = 40) were used in the experiments, weighing between 250 and 400 g at the time of surgery. Experiments were conducted exclusively in male rats to minimize sex related biological variability. Rats were housed in an animal room of 12/12 h light dark cycle, at a temperature of 23 ± 2 °C with food and water available ad libitum. All experiments were conducted in accordance with the regulations of the Animal Welfare Committee of Eötvös Loránd University, in conformity with EU Ethical Rules of Using Animals for Research Purposes (2010/63/EU revising Directive 86/609/EEC) and the Hungarian Act of Animal Care and Experimentation (1998, XXVIII). The experimentation permission was obtained from the Hungarian Scientific Ethics Council for Animal Experiments (license number: PE/EA/00514-6/2022)

### 2.3. LTP Paradigm

Male Wistar rats (*n* = 28, 300–400 g) were anaesthetized with intraperitoneally injected urethane (1.25 g/kg, supplemental doses 0.2–0.5 g/kg bodyweight) and were placed into a stereotaxic apparatus (Kopf Instruments, Los Angeles, CA, USA, Model 902). Body temperature was maintained at 36.5 °C using a heating pad (Supertech, Noida, India, Tmp-5b). Bipolar stimulating electrodes were implanted into the dorsomedial perforant pathway. Stimulating and recording electrodes were constructed from formvar-insulated double strand stainless steel wire (0.005 in OD, California Fine Wire Company, Grover Beach, CA, USA). Vertical tip separation was 0.8 mm, and insulation was restored by Epoxylith. Electrodes were attached into a stereotaxic drive (David Kopf Instruments, Tujunga, CA, USA). First, the vertical position of the stimulating electrode was adjusted on the basis of recording the largest possible amplitude of the slow field potential response (fEPSP) by the hippocampal recording electrode lowered to the upper layer of the hippocampus at the coordinates of CA3. The average coordinate of the perforant pathway was AP −8.1, ML −3.0, DV −3.3 mm according to [30]. After successful positioning of the stimulating electrode, the recording electrode was adjusted vertically so that fEPSP was the highest and it was superimposed by a population spike. The average coordinates of the CA3 recording electrodes were AP −3.0, ML −2.7, DV −3.8. Electrical signals were amplified by a Grass EEG (Grass EEG8B) amplifier, and a CED 1401 data capture system using Signal 4.1 software was applied for data collection and analysis. To inject RIPSYR and NVILQIG reference peptides, a stainless-steel guide cannula was placed into the lateral ventricle (AP −3.9, ML −5.0, DV −4.7) in close proximity to the CA3 region, and a fused silica capillary cut to project 1.00 mm from the bottom of the cannula was used for injection. Peptides were dissolved in artificial cerebrospinal fluid (aCSF) and 7 μL total volume was applied; the speed of injection was 1 μL/min. Three different concentrations (7.5 μM, 75 μM, 750 μM) were tested.

The LTP induction paradigm started by establishing the LTP inducing stimulus amplitude as twice of the stimulus threshold. The LTP-stimulation paradigm consisted of a high-frequency stimulation (HFS; 99 impulses at 400 Hz, 0.3 ms width). The recording lasted 30 min after HFS-LTP induction. For analysis, the magnitude of LTP is expressed as the percent change in the slope of the fEPSP post-HFS as compared to the last five minutes of the pre-injection baseline. The magnitude of post tetanic potentiation (PTP) was calculated as the average fEPSP slope over the 0–3 min period post HFS. Early phase LTP (eLTP) magnitude was calculated as the average fEPSP slope over the 27–30 min period post HFS. Treatment effects on LTP magnitude were compared with animals receiving only aCSF or aCSF + NVILQIG reference peptide. Data were normalized to the baseline values and statistically evaluated by one-way ANOVA with Dunnett’s post-hoc test to compare the effect of different concentration of RIPSYR treatments against the control aCSF group.

### 2.4. Cheeseboard Maze Task

Male Wistar rats (*n* = 8, 300–375 g) were used in a spatial learning paradigm using a cheeseboard maze task. Rats were anaesthetized with isoflurane (3% for induction and 1.5% for maintenance) on a heating pad (Supertech, Tmp-5b) and after positioning in a stereotaxic frame, an incision was made to expose the skull. A stainless-steel guide cannula was implanted into the lateral ventricle under aseptic conditions through a hole drilled in the skull at (AP −3.9, ML −5.0, DV −4.7) and was secured with dental cement. After surgery animals received Baytril (150 μL/kg) subcutaneously once a day for 4 days and lidocaine ointment to relieve pain. Following a one-week recovery period, rats were kept on low diet reducing their weight to 85% of the preoperative weight. Water was available ad libitum. The cheeseboard maze was a 120 cm in diameter circular board with 177 food wells (2.5 cm id), mounted on a rotating support frame at a height of 60 cm above the floor. The start box with a sliding door was used as a starting point for the learning trials and the animals returned to it after successfully obtaining all 3 rewards on the cheeseboard. Animals were separated into two groups, one receiving ACSF (7 μL) on the first day followed by RIPSYR (7 μL, 75 μM) administration the second day and the other group receiving RIPSYR first, followed by aCSF the next day. We followed the learning protocol as outlined by [31,32]; in brief, each daily session included five phases, a pre-probe test (prior rewards memory), a pre-learning rest phase, a learning phase (40 trials), a post-learning rest phase and a post-probe test (recent rewards memory). The 3 food reward locations were the same for each animal on the same day but changed day-to-day along the 40 trials. To avoid animals being guided by smell, the cheeseboard was cleaned and rotated 90 degrees relative to the start box between learning trials. During post and pre-probe trials animals were not rewarded. Memory retention performance was estimated by revisiting the previously learned reward locations during post-probe trial (2 h after learning trials) and pre-probe trial (24 h after learning trials). After the post-probe trials, rats were deeply anesthetized with urethane i.p. (25 *w/v* %, dosage: 1 mL/200 g), and the animals were perfused with PBS (pH 7.4) to remove blood contamination. The brains were quickly removed and were frozen in dry ice-ethanol and stored at −80 °C until further analysis.

### 2.5. Pose Estimation and Behavioral Analysis

DeepLabCut version 3.0.0rc6 [33,34] was used to track 7 key-points (nose, left ear, right ear, body center, body center left, body center right, tail base) in each animal. Four hundred seventy-nine frames were manually labelled taken from 23 videos (95% of labeled frames were used for training, the remaining 5% were used for neural network evaluation). We used a ResNet-50-based neural network [35,36] with a shuffle of 5 and batch size of 8 for 250 number of training epochs. This resulted in a training error of 5.19 and a testing error of 6.4 with a p-cutoff of 0.4 (image size was 1120 by 1080). This network was then used to analyze videos from similar experimental settings. Videos and their tracking data from DeepLabCut were imported into SimBA (Simple Behavioral Analysis) version 2.5.7 [37]. Gaussian smoothing was applied to the tracking data over 200 ms intervals. Pixel/millimeter density values were calculated by using the cheeseboard as a reference, resulting in approximately 0.9 pixel/millimeter value across the videos. As the accuracy of our trained pose estimation neural network in DeepLabCut was sufficient, outlier correction was skipped. Then, the location of the food rewards and the cheeseboard itself were marked with ROIs in the recordings of each trial. For the estimation of learning performance, travel distance in each learning trial was determined from the keypoint tracking of the body center of the animal between leaving and re-entering the start box. ROIs were plotted around the locations of the rewards. For memory retention performances, the number of nose keypoint-ROI overlaps and the percentage of time spent in ROIs of the first three minutes were measured in the pre- and post-probe tests.

### 2.6. Arc Level Measured by Western Blot

We carried out Western blot experiments to study Arc level upon RIPSYR treatment on whole hippocampus of animals (*n* = 20) that were subjected to LTP measurements and RIPSYR administration in three different concentrations. In rats that performed the cheeseboard maze tasks, we included cingulate cortex (CC), which is known to play an important role in remote spatial memory retrieval [38], and cerebellum (CRB) as a control area. Thus, we performed Western blot measurements on CA1, CA3, CC, and CRB of the animals (*n* = 8). Brain samples from whole hippocampus, CA1, CA3, CC, and CRB areas were homogenized in urea/thiourea lysis buffer (LB) (7 M urea, 2 M thiourea, 4% CHAPS, 20 mM Tris, 5 mM magnesium-acetate) supplemented with protease and phosphatase inhibitor cocktails (Sigma-Aldrich, Burlington, MA, USA). Whole hippocampus samples were homogenized using Dounce Tissue Grinder (Pestle B; Sigma-Aldrich, Burlington, MA, USA), centrifuged at 10,000× *g* for 10 min and supernatants were collected. CA1, CA3, CC, and CRB areas were homogenized using a cordless motor (Kimble, Dover, OH, USA) and sample grinding kit (Cytiva, Marlborough, MA, USA) by following the manufacturer’s protocol. Briefly, samples were grinded for 1 min at RT (in 300 μL LB/100 mg of tissue), then centrifuged for 5 min at 10,000× *g* to pellet the resin and debris. Samples were incubated for 30 min at RT with gentle agitation and sonicated three times for 10 s. Then, samples were centrifuged again for 15 min at 10,000× *g*, supernatants were collected. Protein concentration of all samples was measured by 2D-Quant kit (Cytiva, Marlborough, MA, USA). Samples (containing 40 µg protein in the case of whole hippocampus and 50 µg in the case of CA1, CA3, CC, and CRB areas) were diluted with sample loading buffer (0.1 M Tris–HCl pH  =  6.8, 8 *w/v*% SDS, 24 *v/v*% glycerol, 200 mM DTT, 0.2 *v/v*% bromophenol blue) and incubated at 96 °C for 5 min. Proteins were separated on 7.5% acrylamide gel by SDS-PAGE (Bio-Rad Laboratories, Hercules, CA, USA). Then, proteins in the gels were transferred to PVDF membranes (Bio-Rad Laboratories, Hercules, CA, USA) at 20 V for 2 h in a wet-blot system (Bio-Rad Laboratories, Hercules, CA, USA). Membranes were washed with distilled water and stained with Ponceau S stain (0.5 (*w/v*)% in 1 (*v/v*)% acetic acid) to visualize total protein content, after washing with distilled water; they were photographed. Membranes were destained in 200 µM NaOH and 20% acetonitrile and washed again in distilled water, then blocked with 5% BSA solution in Tris-buffered saline with Tween-20 (TBS-T; 20 mM Tris-HCl, 137 mM NaCl, 0.05% Tween-20, pH 7.6) for 1 h at RT. Membranes were stained with primary antibody: anti-Arc (at 1:2000 dilution in blocking solution) (Synaptic System, Göttingen, Germany, Cat # 156-003) at 4 °C overnight and washed in TBS-T. Secondary antibody staining with A647-conjugated anti-rabbit (Jackson ImmunoResearch, West Grove, PA, USA, Cat # 711–605-152) was applied in 1:800 dilution for 2 h at RT. The antibody-labeled membrane was scanned with FLA 5100 scanner (Fujifilm, Tokyo, Japan) with appropriate laser and filter settings and 50-micron resolution. Images were analyzed with ImageJ (version 1.54 g); we performed a two-step normalization method to minimize membrane-to-membrane variance. First, we determined the densitometric values of Ponceau S staining and normalized them to the sum of band intensities on each blot, we carried out the same procedures in the case of antibody-labeled bands. Next, we normalized antibody-labeled bands again to the ‘prenormalized’ Ponceau S staining. Unpaired, two-sided Wilcoxon rank sum-test (*p*  <  0.05) was applied on the normalized fluorescence intensities in R with the ‘wilcox.test’ function of the ‘stats’ package (v4.4.2; [39]) to confirm altered levels of Arc protein. Western blot results were visualized by boxplots created in R using ‘ggplot2’ and ‘ggsignif’ packages [40,41]. In all cases, the lower and upper hinges of boxes correspond to the first and third quartiles (the 25th and 75th percentiles). The upper and lower whiskers extend from the hinge to the largest and smallest values no further than 1.5 * IQR from the hinge, respectively (where IQR is the inter-quartile range, or distance between the first and third quartiles). Data beyond the end of the whiskers are called “outlying” points and are plotted individually.

### 2.7. Peptide Coupling to Magnetic Beads

PureProteome 1.0 µm Carboxy FlexiBind Magnetic Bead System (Merck Millipore, Burlington, MA, USA) was used to identify binding partners of RIPSYR peptide in synaptosome lysates. First, RIPSYR peptide was covalently crosslinked to carboxylated microparticles. Bead slurry (200 µL/sample) was washed three times with activation/coupling buffer (50 mM 2- (N-morpholino) ethanesulfonic acid (MES); pH 6, 0.01% Triton X-100). Next, 2 mg of 1-ethyl-3- (-3-dimethylaminopropyl) carbodiimide hydrochloride (EDC, Sigma-Aldrich, Burlington, MA, USA) and 2 mg of N-hydroxysuccinimide (NHS, Sigma-Aldrich, Burlington, MA, USA) dissolved in activation/coupling buffer were added to the beads and vortexed. The tubes were incubated with continuous mixing for 15 min at RT. The supernatant was removed from the beads and washed with activation/coupling buffer. Then, 2 mg of peptide dissolved in activation/coupling buffer was added, and the beads were incubated with continuous mixing for 2 h at RT. The beads were washed with activation/coupling buffer, vortexed, and the supernatant was removed. Next, beads were washed with quenching buffer (25 mM Tris-HCl, 130 mM NaCl, 2.7 mM KCl, 0.01% Triton X-100, pH 8) and incubated for 30–60 min at RT. Lastly, beads were washed again three times with quench buffer and were stored at 4 °C.

### 2.8. Recombinant Arc Protein Expression and Purification

The cDNA of Arc N-lobe domain (208–278) was amplified by PCR, then digested and cloned into the BamHI/XhoI sites of pGEX-4T-1 vector (Sigma-Aldrich, Burlington, MA, USA). The construct was verified by Sanger sequencing. E. coli BL21-DE3 were transformed and grown at 37 °C with rigorous shaking in 50 mL LB media, 100 μg/mL ampicillin for 3 h. 15 mL preculture was then used to inoculate 1 L media, then incubation continued until an OD600 of 0.9–1 was reached. To induce protein expression, 1 mM isopropyl b-D-1-thiogalactopyranoside (IPTG) was added, then the temperature was decreased to 18 °C. After 16–18 h of incubation, cells were harvested by centrifugation (4500 rpm, 10 min, 4 °C). Cells were re-suspended in PBS (137 mM NaCl, 2.7 mM KCl, 10 mM Na2HPO4, 1.8 mM KH_2_PO_4_, pH 7.4) and lysed by sonication. The lysate was clarified by centrifugation (14 500 rpm, 20 min, 4 °C) and filtered. The supernatant was applied to a GSTrap Fast Flow column (Cytiva, Marlborough, MA, USA), pre-equilibrated and washed with PBS. GST-Arc N-lobe was eluted with PBS supplemented with 20 mM L-Glutathione reduced. The eluted fractions were dialyzed against PBS overnight, then cleaved by thrombin protease for 3–4 h at room temperature. The cleaved Arc N-lobe was separated from the GST tag by a second GST purification step in similar conditions to those described above. Finally, the target protein was further purified by a size-exclusion chromatography with a Superdex75 10/300 GL column (Cytiva, Marlborough, MA, USA). Protein molecular weight and purity were confirmed by SDS-PAGE. Protein samples were snap frozen with liquid nitrogen and stored at −80 °C until analysis.

### 2.9. Synaptosome Preparation

Male Wistar rats (*n* = 4, 350–400 g) were deeply anesthetized with urethane i.p. (25 *w/v* %, dosage: 1 mL/200 g) and the animals were perfused with PBS (pH 7.4) to remove blood contamination. Their brains were quickly removed and rinsed in ice-cooled PBS. First, the isolated brain was placed on a pre-cooled Petri dish and cut into two halves in the mid-sagittal plane. The cerebral cortices from both hemispheres were separated, then the hippocampi were isolated on ice. The subcellular fractionations were performed immediately after the dissections. The fraction of synaptosomes was prepared following the protocol published by [42,43]. Briefly, hippocampi were homogenized in a homogenization solution (320 mM sucrose, 0.1 mM CaCl_2_, 1 mM MgCl_2_) supplemented with protease and phosphatase inhibitor cocktails (Sigma-Aldrich, Burlington, MA, USA) and homogenized with a small clearance pestle Dounce tissue grinder (Kimble, Dover, OH, USA) at 4 °C with pre-cooled solution and equipment. The sucrose concentration was adjusted to 1.25 M; then, 1 M sucrose, 0.1 mM CaCl_2_ solution was layered on top of the homogenate, and the samples were ultracentrifuged at 100,000× *g* for 3 h in a SW-40 swinging bucket rotor. After ultracentrifugation, the synaptosome pellet was solubilized in wash buffer (50 mM Tris Cl, 300 mM NaCl, pH 7.4) supplemented with protease and phosphatase inhibitors and sonicated three times for 30 s. Then, Triton X-100 was added to the samples to a final concentration of 1%, and they were incubated on a tube roller for 60 min at 4 °C. The samples were centrifuged at 16,000× *g* for 30 min at 4 °C, and supernatants were collected.

### 2.10. Protein Capturing by RIPSYR Peptide

Synaptosome samples (200 µL) or recombinant Arc N-lobe were added to RIPSYR peptide-coupled and uncoupled control beads and incubated for 2 h at 4 °C with constant mixing. Then, the supernatant was removed, and the beads were washed three times with ice-cold wash buffer, ensuring a total wash time of 30 min, with samples kept on ice for 3–5 min between washes. Finally, the supernatant was completely aspirated. The captured proteins were then eluted using the following method: 25 µL of elution buffer (2% SDS in wash buffer) was added to beads and mixed. The mixture was incubated for 5 min at RT. Next, the tubes were placed on a magnetic rack and the supernatant was then transferred to a fresh tube. This elution step was repeated once more, resulting in a total of two elutions. Eluted samples were sent to mass spectrometry-based (MS) protein identification. Using the STRING database (version 12.0, string-db.org (accessed on 17 January 2025) functional enrichment analysis was performed to identify overrepresented gene ontology molecular function terms within the identified binding partners.

### 2.11. Liquid Chromatography–Mass Spectrometry (LC-MS)

The protein content of the affinity isolated samples was treated with trypsin using the S-Trap protocol (ProtiFi LLC, Fairport, NY, USA). Briefly, proteins were reduced using Tris (2-carboxyethyl)phosphine (TCEP), free sulfhydryls blocked by S-methyl methanethiosulfonate (MMTS) followed by proteolysis using trypsin (1 µg/sample, 2 h at 47 °C). The resulting peptide mixtures were labeled with the tandem mass tag (TMT) 6plex isobaric labeling tags (Thermo Fisher Scientific, Waltham, MA, USA), mixed in 1:1 ratio and loaded onto C18 EvoTips (Evosep Biosystems, Odense, Denmark) for LC-MS/MS analysis. Reversed-phase separation of the peptides was performed using an Evosep One HPLC (Evosep Biosystems, Denmark) applying the „30 SPD” 44-min gradient followed by data dependent acquisition using an Orbitrap Fusion Lumos Tribrid (Thermo Fisher Scientific, Waltham, MA, USA) instrument. High resolution MS2 data were generated from the top 10 most precursor ions. Peptide identification and quantitation were done using the Proteome Discoverer software (v2.5). Peptides and proteins were identified with Sequest HT search engine using the rat subset of the UniProt protein database (downloaded 2 November 2020, 35,849 sequences). Relative quantitation was performed using the TMT reporter ion signal-to-noise (S/N) values extracted from the HCD spectra. Only unique peptides were considered for pairwise ratio based comparison of the sample groups (acceptance parameters: Sequest HT Xcorr > 1, precursor ion co-isolation threshold in MS1 < 30%, average TMT reporter S/N in HCD spectra > 10). Proteins identified with high confidence showing |log2FoldChange| > 1.3 were accepted as differently expressed.

### 2.12. In Silico Peptide Docking

We carried out the in silico docking of RIPSYR peptide on the GluA2 ligand binding domain (LBD) dimer using 2al5 [44], and Gabra1 using 6dw1 [45], and Gabra2 using 9crv [46] structures from Protein Data Bank (PDB). However, Gabra2 did not have a crystal structure in Rattus norvegicus, only in Homo sapiens. As a positive control, we also performed the in silico docking of RIPSYRYR peptide on the Arc N-lobe using 6tno structure [27] to have a base for comparing docking results. We have utilized LightDock which is a protein–protein, protein–peptide and protein–DNA docking framework based on the glowworm swarm optimization algorithm [47,48]. First, we discarded the ligand, carbohydrate, and water molecules from the PDB structures, then set up the simulation with ignoring terminal oxygen and hydrogen atoms. Then, we ran the simulation with 100 steps and ‘fastdfire’ scoring function. Next, we generated 200 models predicted in the 100th step, and clustered the generated models for each swarm, and selected the representative models of the top 5 scored clusters for molecular dynamics and enthalpy estimation.

### 2.13. Molecular Dynamics and Enthalpy Estimation

We performed molecular dynamics simulation with GROMACS software (version 2025.1) [49] on the 6tno and docked structures to gain insights on the stability of complexes. Additionally, we carried out enthalpy estimation of the binding of peptides to proteins using molecular mechanics energy combined with the generalized Born and surface area continuum solvation (MM-GBSA) method [50,51]. First, PDB files were prepared and hydrogen atoms were added; in all cases, AMBER ff99SB protein force field [52] and TIP-3 point water model [53] were selected. Then, water and NaCl molecules were added (in 150 mM concentration) to neutralize the charge of the system. The energy of the system was minimized in several rounds using, first, steepest descent, then, conjugate gradient methods; minimization was converged when the maximum force was smaller than 100. Next, we performed molecular dynamics with 1000 frames and 0.001 ps time steps to obtain simulations with lengths of 5000 ps. We have confirmed the stability of the complexes by analyzing the root mean square deviation (RMSD) and root mean square fluctuation (RMSF) of complexes. In addition, we estimated the number of H-bonds throughout the simulation between the protein and peptide pairs. Then, we estimated enthalpy changes upon complex formation in silico. We applied gmx_MMPBSA tool which is based on AMBER’s MMPBSA.py script aiming to perform end-state enthalpy calculations with GROMACS files [54,55].

## 3. Results

### 3.1. RIPSYR Effect on High Frequency Stimulation-Induced LTP

**Figure 1:**
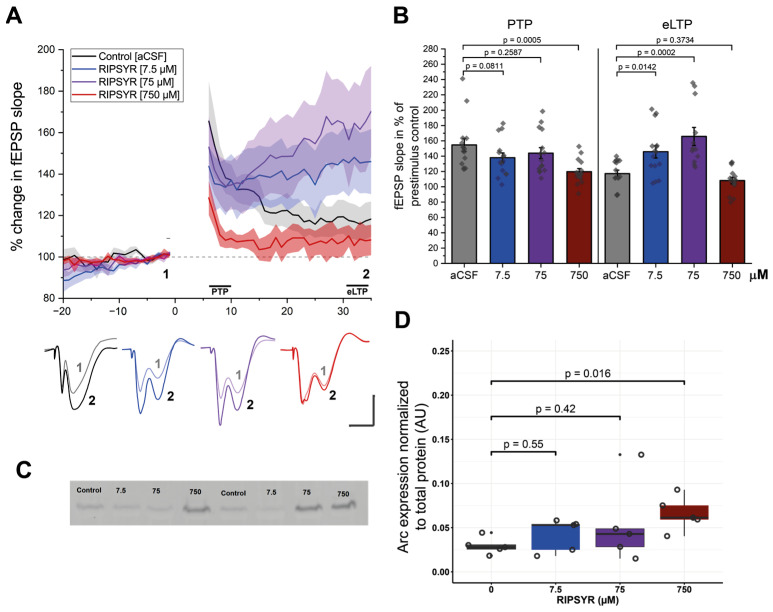
LTP measurements in RIPSYR treated animals. (**A**) The time course of the mean fEPSP slope after HFS induced LTP following RIPSYR administration. Gray dashed line denotes baseline fEPSP, shaded area denotes SEM, solid lines denote the initial PTP phase and the following eLTP. Lower panel shows representative traces of fEPSPs, numbers 1–2 correspond to baseline and eLTP as indicated in the upper panel. Scale bars indicate 1 mV and 5 ms. (**B**) Summary of the magnitude of potentiation at PTP and eLTP in all treatment groups, with individual datapoints (One-way ANOVA with Dunnett’s post-hoc was applied for comparison. All values are reported as mean ± SEM.) (**C**) Representative image of anti-Arc labeled membrane. (**D**) Arc level normalized to total protein content in aCSF (0 µM) and RIPSYR treated animals in ascending concentrations, with individual datapoints (*n* = 20; unpaired, two-sided Wilcoxon rank-sum-test was applied for statistical comparison).

Modulating AMPAR mobility is known to affect long-term potentiation in the hippocampus in vitro and in vivo [56]. In the present work, we investigated whether LTP is altered in vivo by the TARPγ2—derived RIPSYR peptide. LTP at perforant path-to-CA3 was induced by high frequency stimulation (HFS). All three concentrations of RIPSYR peptide treatment decreased the initial post-tetanic potentiation (PTP) phase of LTP. Analysis of the PTP phase revealed a significant difference between groups, F (3, 56) = 4.86, *p* < 0.001, α = 0.05. Dunnetts post-hoc test indicated that the 750 µM RIPSYR group (Figure 1A,B; 119.63 ± 4.12%, *p* = 0.0005) reached statistical significance compared to the control group (154.70 ± 8.42%). At the eLTP phase analysis revealed a significant difference between groups, F (3, 53) = 12.55, *p* < 0.001, α = 0.05. Dunnetts post-hoc test indicated that the low and medium concentrations of the peptide significantly increased the fEPSP slope (RIPSYR: 7.5 μM: 145.7 ± 8.07%, *p* = 0.0142; 75 μM: 165.7 ± 11.71%, *p* = 0.0002) compared to the controls (117.2 ± 4.55%) (Figure 1A,B). In response to the high dose, fEPSP slope decreased significantly in the PTP phase and rapidly returned to near to the baseline and stayed at the baseline value along the rest of the recording (108.18 ± 4.16%, *p* = 0.16) (Figure 1B). To rule out the possibility that the delayed fEPSP changes were due to slow tissue penetration of RIPSYR, a control experiment was carried out (*n* = 3) in which high-frequency stimulation was initiated 20 min after RIPSYR injection. The temporal dynamics of LTP remained comparable across both delayed and non-delayed groups, indicating that diffusion-related factors are unlikely to account for the effects of RIPSYR on LTP (Supplementary Figure S1). We applied the Arc binding peptide NVILQIG (750 μM; *n* = 5) targeting the capsid formation surface of Arc. No significant changes in fEPSP slope were observed during PTP or eLTP phases (Supplementary Figure S1), suggesting, that the observed effect is RIPSYR specific.

### 3.2. Arc Levels Are Elevated in RIPSYR Treatment

To investigate if Arc protein levels are changed as a result of RIPSYR treatments in LTP, we performed Western blot experiments on whole hippocampus of rats (*n* = 20) (Figure 1C,D). In the 7.5 µM and 75 µM RIPSYR treated group, we did not find significant changes in expression levels of Arc (fold change (FC): 1.42 and 1.82, respectively; *p*-value: 0.55 and 0.42, respectively). However, in the 750 µM RIPSYR treated group Arc expression increased significantly (FC = 2.45, *p*-value = 0.016).

### 3.3. RIPSYR Treatment Affects Memory Retention on Cheeseboard

**Figure 2:**
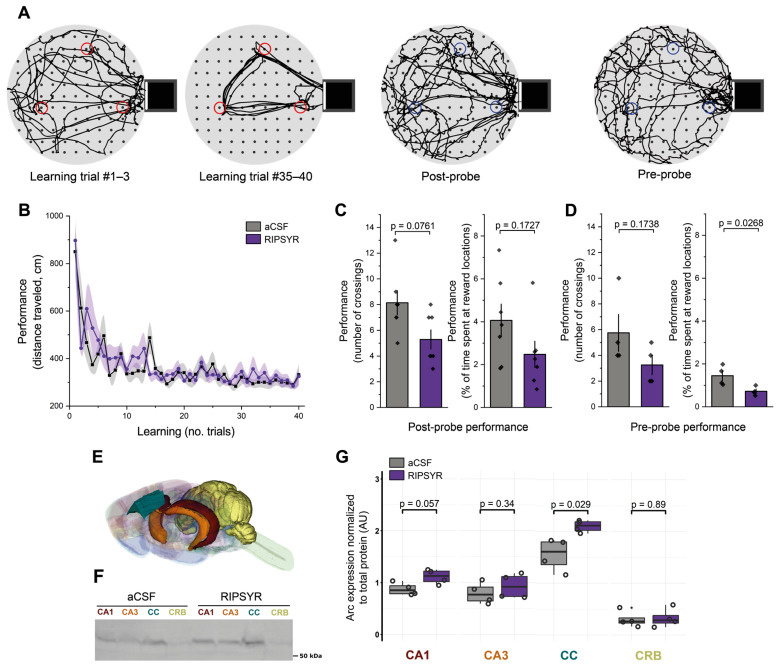
Reward location learning on the cheeseboard maze. (**A**) Representative examples of an animal’s path during the learning trials (red circles: reward locations with food) and during the probe sessions (blue circles: previously learnt reward locations without food). (**B**) Learning performance of aCSF and RIPSYR treated animals during learning trials (means ± SEM). (**C**,**D**) Memory retention performance during post- and pre-probe tests, showing the number of crossings and the percentage of dwell time of animals spent at reward locations, with individual datapoints. (**E**) Color coded brain regions analyzed with Western blot, red: CA1; orange: CA3; blue: cingulate cortex; yellow: cerebellum. The illustration was constructed from the ‘Waxholm Space Atlas of the Sprague Dawley Rat Brain v4’ [57], available under the licence CC-BY-SA 4.0 (https://creativecommons.org/licenses/by-sa/4.0/) at https://www.nitrc.org/projects/whs-sd-atlas (accessed on 6 March 2025). (**F**) Representative image of anti-Arc labeled membrane. (**G**) Arc expression normalized to total protein content in control and RIPSYR (75 μM) treated animals in several brain regions, with individual datapoints (*n* = 8; unpaired, two-sided Wilcoxon rank-sum-test was applied for statistical comparison).

To test the effect of RIPSYR on learning and memory retention, we used the cheeseboard maze task (Figure 2A), a hippocampus dependent spatial memory paradigm. Before the learning trials rats were administered RIPSYR peptide or aCSF by i.c.v. injection. The first group of the animals first received aCSF and the other group first received RIPSYR. The learning performance of RIPSYR treated rats during the learning trials was not statistically different between aCSF-first and RIPSYR-first groups (linear mixed model, *p* = 0.1935) (Figure 2B). Unifying all RIPSYR and all aCSF treated data without respecting the application order, we did not obtain significant difference in the learning process (RIPSYR, *p*-value: 0.0686; aCSF, *p* = 0.0852). We tested the animals’ memory retention performance by measuring the number of crossings and the time spent at previously learned food locations at 2 h after learning (post-probe) or 24 h after (pre-probe of the next day). Animals treated with RIPSYR scored lower in all measured parameters of memory retrieval. In the post-probe assessments, RIPSYR administration resulted in reduction in the number of crossings (Figure 2C) (RIPSYR vs. aCSF, *n* = 14, mean ± SEM: 5.29 ± 0.75 vs. 8.14 ± 0.94, paired sample t-test, *p* = 0.0761, d = −1.27 (95% CI: [−2.42, −0.13]) and a reduced dwell time percentage (RIPSYR vs. aCSF, *n* = 14, mean ± SEM: 2.48 ± 0.61 vs. 4.06 ± 0.76, paired sample t-test, *p* = 0.1727, d = −0.87 (95% CI: [−1.97, 0.23]), but these differences did not reach statistical significance. However, 24 h later, in the pre-probe session assessment, RIPSYR-treated rats showed a reduced number of crossings relative to control animals (RIPSYR vs. aCSF, *n* = 8, mean ± SEM: 3.25 ± 0.75 vs. 5.75 ± 1.44, two sample t-test *p* = 0.1738, d = −1.09 (95% CI: [−2.58, 0.39]) and a significantly reduced dwell time score compared to the control condition (RIPSYR vs. aCSF, *n* = 8, mean ± SEM: 0.72 ± 0.10 vs. 1.45 ± 0.23, two sample t-test, *p* = 0.0268, d = −2.06 (95% CI: [−3.78, −0.35]), suggesting an impairment in retrieval of previously learned reward locations (Figure 2D). RIPSYR induced a decreasing tendency of number of crossings and dwell time of previously learnt reward locations. The significant decrease in dwell time at the 24 h pre-probe had a large effect size, supporting a meaningful impairment in memory retention. Post-hoc power analysis shows that across our metrics the statistical power ranged from low to moderate (26% to 68%), a consequence of the relatively constrained sample sizes.

### 3.4. Arc Levels Are Increased upon RIPSYR Treatment in the Cingulate Cortex of Rats in Cheeseboard Maze Tasks

We used the Western blot technique to study Arc levels upon RIPSYR treatment on the CA1 and CA3 region of the hippocampus, cingulate cortex (CC) and cerebellum (CRB) brain regions of the animals (*n* =  8) that performed cheeseboard maze task (Figure 2E). In the CA3 and CRB, we did not find significant change in the level of Arc (FC: 1.17 and 1.08; *p*-value: 0.34 and 0.89, respectively). In the CA1 region, Arc level slightly increased, however this did not reach statistical significance (FC = 1.27, *p*-value = 0.057). In the CC, Arc level increased significantly (FC = 1.35, *p*-value = 0.029) in response to RIPSYR administration (Figure 2F–G).

### 3.5. Putative Binding Partners of RIPSYR

**Figure 3:**
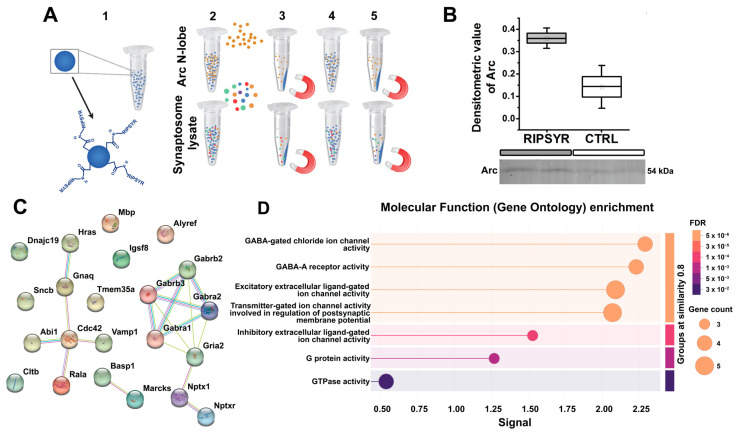
Revealing binding partners of RIPSYR peptide in hippocampal synaptosome proteome. (**A**) Schematic of magnetic bead-based assay to probe RIPSYR binding to Arc N-lobe and synaptosome proteins. (**B**) Validation of Arc N-lobe binding to RIPSYR-coupled beads after magnetic bead assay by Western blot. (**C**) STRING network of binding partners identified by MS that had at least 1.3-fold higher abundance in RIPSYR coupled beads. (**D**) STRING functional enrichment results ranked by signal strength.

We performed a magnetic bead-based assay to establish RIPSYR peptide binding to Arc N-lobe and synaptic proteins. We covalently coupled RIPSYR peptide onto carboxy-modified beads, and precipitated proteins that are bound to the beads (Figure 3A). We have not found Arc among the RIPSYR binding partners measured by MS possibly due to low basal level [58]. Thus, we applied an antibody-based approach, and we could detect recombinant Arc N-lobe binding to RIPSYR-coupled beads by Western blot (Figure 3B). Then, we probed synaptic proteins prepared from hippocampal synaptosome lysate; we quantified peptides derived from 462 proteins with LC-MS with abundance ratios (RIPSYR-coupled bead/blank bead) between 0.48 and 2.59 (Supplementary Table S1). We found neurite outgrowth-related proteins, such as Basp1 (abundance ratio = 2.59), Marcks (abundance ratio = 1.82), Cdc42 (abundance ratio = 1.71), and several ion channel subunits, such as Gabra2 (abundance ratio = 1.503), Gabra1 (abundance ratio = 1.397), Gabrb2 (abundance ratio = 1.344), GluA2 (abundance ratio = 1.328), Gabrb3 (abundance ratio = 1.316) that had at least a 1.3-fold higher abundance in peptide coupled beads compared to blank beads (Figure 3C). These potential interactions were obtained under specific conditions, and additional experimental validation remains necessary to confirm RIPSYR binding partners. Results from the functional enrichment tool of STRING suggest that the protein-protein interactome is highly related to ion channel activity (Figure 3D). Ranking by signal strength indicates association with GABA-A receptors and GABA-gated chloride ion channels, followed by excitatory extracellular ligand-gated ion channel activity. These imply that RIPSYR peptide potentially influences the balance between glutamatergic and GABAergic signaling, possibly affecting overall network activity by modulating neuronal excitability. All binding partners of RIPSYR are able to modulate plasticity and learning so their contribution to the physiological effects of RIPSYR cannot be excluded, particularly, in the case of high concentration application of RIPSYR.

### 3.6. In Silico Peptide Docking and Estimation of Interaction Energies Suggest Possible GluA2 LBD Dimer Binding of RIPSYR Peptide

We performed a magnetic bead-based assay to probe RIPSYR peptide binding to synaptic proteins. Using covalently coupled RIPSYR peptide onto carboxy-modified beads, RIPSYR bound proteins were precipitated and identified by MS. We successfully confirmed Arc N-lobe binding to bead-coupled RIPSYR. Because RIPSYR is a relatively short peptide, the MS analysis of RIPSYR-bound protein lysates yielded a high number of binding partners. Looking for proteins that might be also involved in the action of RIPSYR on synaptic plasticity and learning, we selected GluA2 subunit of AMPAR, and Gabra1 subunit of GABA receptor. Next, we performed in silico docking by LightDock framework [47,48] to determine possible binding regions of RIPSYR.

**Figure 4:**
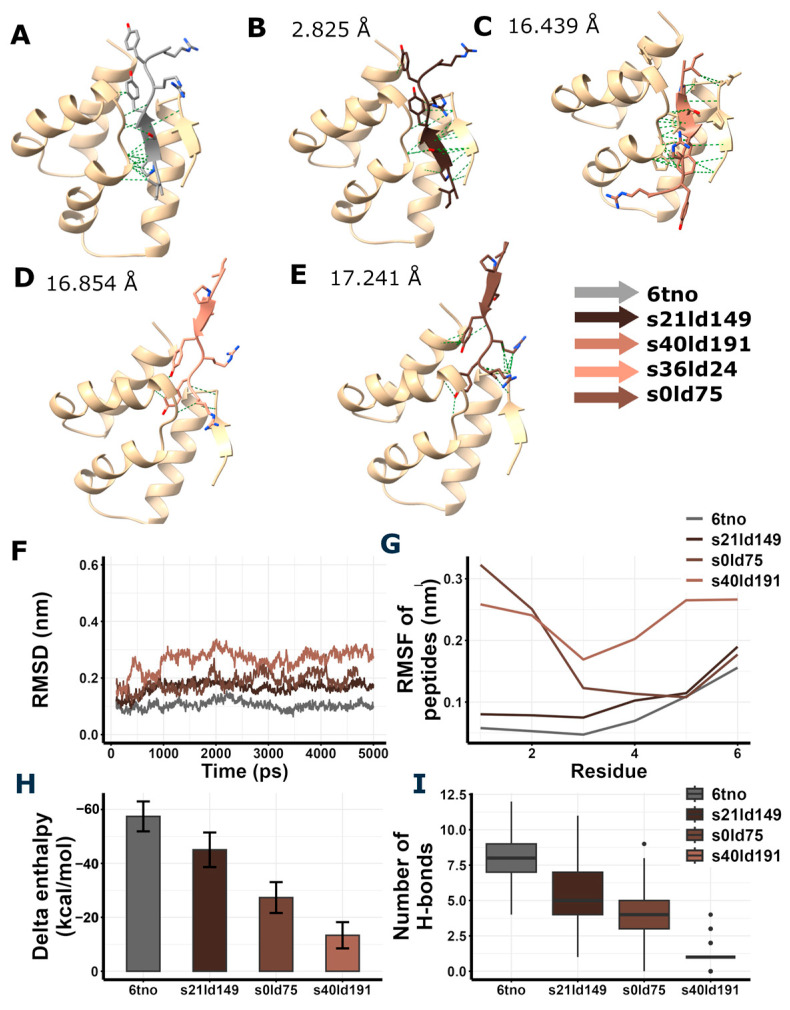
Redocking of RIPSYRYR to Arc N-lobe and molecular dynamics and enthalpy estimation of Arc N-lobe in complex with RIPSYRYR (6tno). (**A**) Crystal structure of Arc N-lobe in complex with RIPSYRYR peptide (6tno). (**B**–**E**) Models with the highest docking scores are shown; root mean square deviation (RMSD) values of RIPSYRYR backbone C atoms between 6tno and redocked models are indicated., models retrieved from redocking RIPSYRYR to Arc N-lobe. (**F**) Root mean square deviation (RMSD) of the backbone C atoms of different models throughout the simulation. (**G**) Root mean square fluctuation (RMSF) of the backbone C atoms of peptides in different models. (**H**) Delta enthalpy changes upon complex formation in different models. (**I**) Number of H-bonds between studied proteins and peptides throughout the simulation.

As a reference, we performed the in silico docking of RIPSYRYR peptide on the Arc N-lobe using 6tno structure [27], where the Arc N-lobe is in complex with RIPSYRYR, to provide a basis for comparing docking results (Figure 4) Then, we carried out the in silico docking of RIPSYR peptide on the GluA2 ligand binding domain (LBD) dimer using 2al5 [44], Gabra1 using 6dw1 [45], and Gabra2 using 9crv [46] structures from PDB. We note here that the 9crv model is based on the protein sequence of Homo sapiens.

Among the highest score models of redocking of RIPSYRYR on Arc N-lobe (Table 1, Figure 4), s21ld149 was found to be the best match to the original 6tno structure, with a root mean square deviation (RMSD) score of 2.825 Å between the C atoms of RIPSYRYR backbone. The root mean square deviation (RMSD) and root mean square fluctuation (RMSF) of the backbone C atoms characterizes the stability of the models. The RMSD of Arc N-lobe (Figure 4F) and RMSF of the peptide (Figure 4G) in 6tno crystal structure showed the lowest values during the simulation indicating high stability. The RMSD and RMSF in s21ld149 were the closest to 6tno curve, further confirming it is the closest match to 6tno structure. The predicted number of H-bonds and estimated enthalpy changes upon complex formation characterize the interaction between peptides and proteins. The delta enthalpy (dH; Figure 4H) and the number of H-bonds (nB; Figure 4I) in 6tno crystal structure were the most favorable throughout the simulation (dH = −57.43 ± 5.54; nB = 7.82 ± 1.42, as mean ± sd) and s21ld149 (dH = −45.05 ± 6.42; nB = 5.57 ± 1.74, as mean ± sd) was the most similar to 6tno in agreement with previous observations. Therefore, the closest match to the reference model could be determined based on the RMSD, RMSF, enthalpy change, and the number of H-bonds.

**Figure 5:**
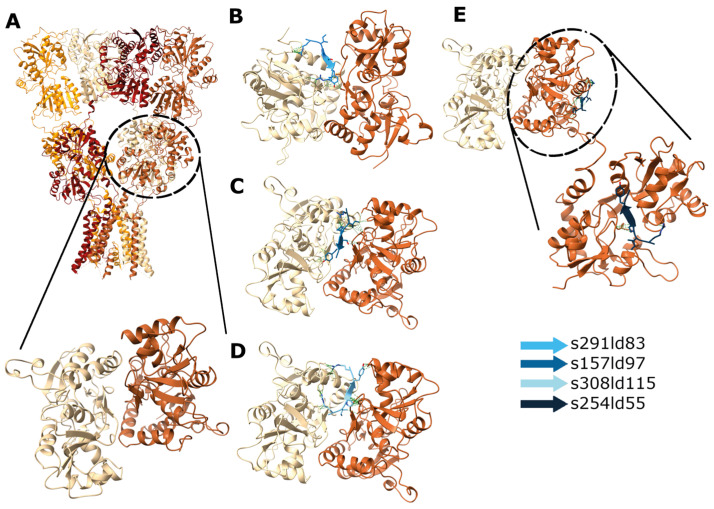
Docking of RIPSYR peptide to GluA2 LBD dimer. (**A**) Structure of GluA2 homotetramer (upper panel; 3KG2; [52] and GluA2 LBD dimer (lower panel; 2al5). Among the highest scored models obtained from peptide docking, (**B**–**D**) three models had the peptide between the two ligand binding domains and (**E**) one model near the ligand binding region (the colors of the peptides indicate different models).

**Figure 6:**
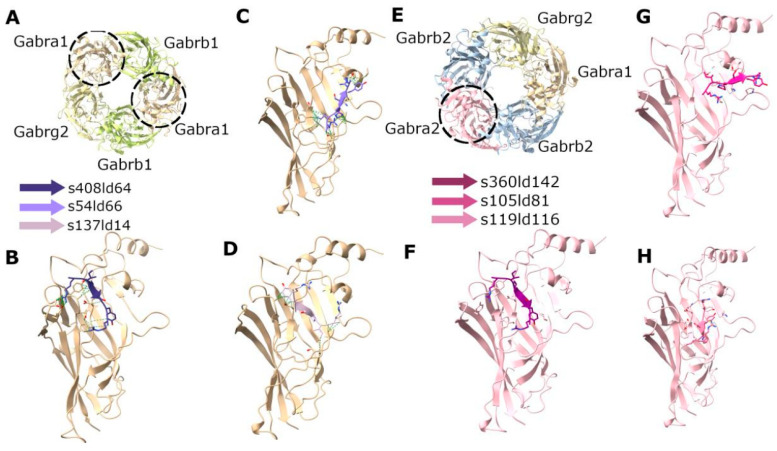
Docking of RIPSYR peptide to the extracellular domains of Gabra1 and Gabra2. (**A**) Structure of rat GABA receptor complex (6dw1). (**B**–**D**) The three highest scored models of Gabra1 and RIPSYR complex. (**E**) Structure of human GABA receptor complex (9crv). (**F**–**H**) The three highest scored models of Gabra2 and RIPSYR complex (the colors of the peptides indicate different models).

In the case of docking RIPSYR to GluA2 LBD dimer, we found that, among the highest scored models, most of them had the peptide binding site between the two ligand binding domains (Figure 5B–D) and on one model was located near the glutamate binding region (Figure 5E). In the case of docking RIPSYR to Gabra1 and Gabra2, we found that the highest scored models had the peptide docked on the inner surface of the chloride channel (Figure 6). The human sequence of Gabra2 has 3 SNPs compared to the rat sequence besides the signal region, however, these are not near the docked binding surfaces.

Since the Arc-RIPSYRYR model with the highest docking score was not the closest match to the original structure, it was necessary to obtain further insights into the acquired models to provide a more reliable prediction regarding RIPSYR interactions with GluA2, Gabra1 and Gabra2. Thus, we performed a molecular dynamics simulation using GROMACS software [49] on the docked structures to compare the stability of the models. Additionally, we carried out the estimation of enthalpy changes upon interactions between peptides and proteins by gmx_MMPBSA tool [54,55].

**Figure 7:**
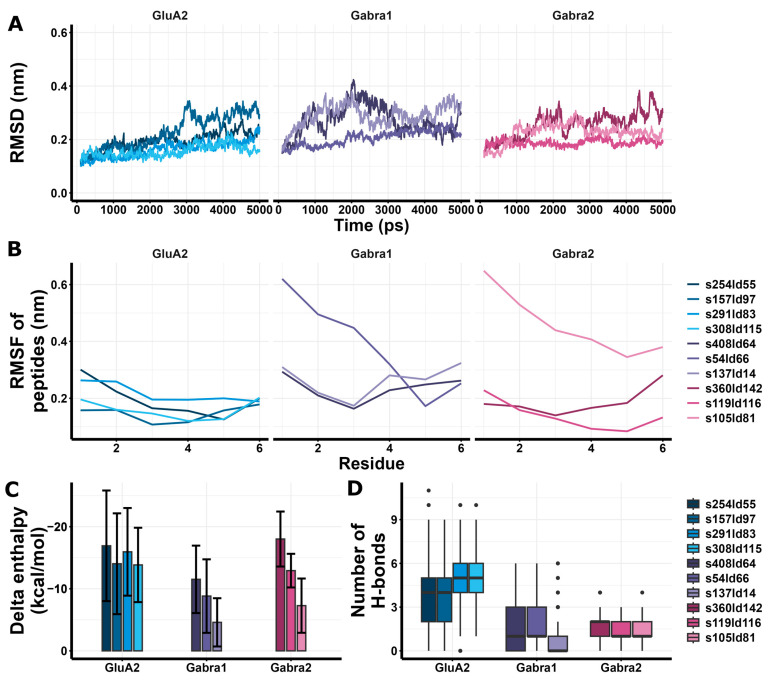
Molecular dynamics and enthalpy estimation of docking models of RIPSYR to GluA2 LBD dimer, Gabra1 and Gabra2. (**A**) Root mean square deviation (RMSD) of the backbone C atoms of different models throughout the simulation. (**B**) Root mean square fluctuation (RMSF) of the backbone C atoms of peptides in different models. (**C**) Delta enthalpy changes upon complex formation in different models. (**D**) Number of H-bonds between studied proteins and peptides throughout the simulation (black circles represent outliers).

GluA2 models had similar RMSD values to Gabra1 and Gabra2 (Figure 7A), and GluA2 models had lower peptide RMSF values compared to Gabra1 (Figure 7B). In addition, GluA2 models showed more favorable enthalpy changes than Gabra1 and Gabra2 models except for s360ld142 (Figure 7C). Regarding H-bonds, GluA2 models showed a larger number of H-bonds compared to Gabra1 and Gabra2 models (Figure 7D). Based on these results, RIPSYR binding to Gabra1 are less favorable compared to GluA2 binding.

## 4. Discussion

The consolidation of input-driven synaptic molecular changes is the leading model of learning and memory at the molecular level [59]. Regulation of AMPAR anchoring to the PSD95 scaffold is a critical step in the modulation of the strength of synaptic transmission [60]. Auxiliary proteins, such as TARPs, help AMPAR anchoring to the synaptic scaffold [16], while Arc regulates AMPAR detachment from PSD95 [21]. A hexapeptide, RIPSYR—designed to block the binding of Arc to TARPγ2—was developed and validated in in vitro studies [20,21]. However, its effects in vivo on physiological processes such as plasticity and spatial learning are not clear. In this paper, we report our findings on the in vivo effects of RIPSYR peptide on synaptic plasticity and spatial memory consolidation and its off-target binding partners.

First, we examined the effects of RIPSYR administration on plasticity, using induced LTP in the CA3 of rats. In our study, the i.c.v. administration of RIPSYR had effects on LTP in a concentration-dependent manner. Compared to control aCSF injections, the fEPSP slope in PTP phase slightly decreased independently of the RIPSYR concentration, but only reached a statistically significant level at the highest dose. In contrast, in eLTP, a significant increase in the fEPSP slope was apparent. Due to concerns regarding variability introduced by randomizing short peptides [61], a scrambled peptide control was not employed in the experimental design. Sequence randomization can significantly modify physicochemical properties, thereby complicating the interpretation of the results. Instead, we employed NVILQIG, a peptide targeting the oligomerization site of Arc that is distinct from the RIPSYR binding interface. This approach resulted in no significant effects, suggesting that the physiological changes observed following RIPSYR treatment are likely sequence-specific. However, based on the available data, we cannot conclusively attribute these effects to a single protein-binding interaction. We hypothesize that the putative effect of RIPSYR on the inhibition of AMPAR detachment from the synaptic scaffold evokes the observed increase in synaptic strength. As AMPAR mobility declines, the AMPAR anchoring increases, it results in enhanced fEPSP during eLTP. It is known that expression of Arc is regulated by neuronal activity, and that it has low basal expression [62]. It has been revealed that Arc level is increased during LTP [63]. In consideration of this, we examined how RIPSYR treatment influences Arc levels during eLTP. Our data suggest that Arc level increases in a dose-dependent manner after LTP stimulation in the presence of RIPSYR. The increase was only a tendency at low and medium doses, but it became statistically significant at the highest dose. This suggests that when the Arc level is low or missing at the initial phase, RIPSYR may affect PTP through aspecific binding partners or other physicochemical effects. The enhancement of fEPSP and the subsequent increase of Arc level by RIPSYR during eLTP were unknown. We hypothesize that, in the case of low and medium dose RIPSYR treatment, the elevation of Arc availability in the synapses was moderate and the LTP-induced Arc expression was within the physiological range. However, the high dose 750 µM RIPSYR treatment elevated the Arc level to nearly 2.5-fold compared to baseline and resulted in a decrease of PTP and a complete block of eLTP. A critical aspect of our findings is the dose-dependent, biphasic effect of RIPSYR on LTP. It should be noted that this biphasic effect may stem from subacute physicochemical stress or cytotoxic mechanisms. Several biophysical phenomena could be responsible for this observation, such as peptide aggregation, altered osmolarity or membrane disruptions that should be addressed in future in vivo and in vitro studies. In sum, the highest concentration of RIPSYR (750 µM) induced an adverse effect on LTP and Arc level, while lower doses had a potentiating effect on LTP accompanied by a moderate increase of Arc level. Based on our results, RIPSYR was used in 75 µM concentration in the following spatial learning study.

Although LTP is a frequently applied model to study synaptic plasticity, it does not exhaustively characterize the learning process, because high-frequency stimulus is required for its induction, which is not apparent in physiological circumstances. Thus, it was important to follow up with the hippocampus-dependent cheeseboard task to determine whether synaptic alterations induced by RIPSYR translate into deficits in spatial memory. In the performed cheeseboard spatial learning test, the learning curve was not influenced by RIPSYR. In contrast, memory retrieval performance after 24 h declined, but not after 2 h, indicating that memory consolidation is involved in the mechanism. While our findings suggest an impairment in memory consolidation induced by RIPSYR, it is important to interpret these results cautiously due to the low-to-moderate statistical power associated with our limited sample sizes. To enhance interpretability and strengthen conclusions, further studies with larger sample sizes are needed. Notably, our findings align with the results of El Oussini and colleagues [64]. In their study, immobilizing cell surface AMPARs in the hippocampus of mice using antibody-based cross-linking also showed that CA3 synaptic plasticity was not necessary to encode the spatial memory rule in a delayed spatial alternation task. In contrast, immobilization of AMPARs before or post-encoding led to an apparent forgetting of the spatial rule. While targeting AMPAR mobility by a different but conceptually related approach, their core observation is in agreement with our results that inducing AMPAR immobility results in loss of memory consolidation but not encoding. Their work further clarified the possible mechanism of the deficit in memory consolidation by linking AMPAR immobilization to altered hippocampal ripple physiology. In 1989, Buzsáki [65] published a seminal work in which he proposed sharp wave-ripple (SWR) complexes as key events in the induction of LTP during offline brain states. This proposal posited that these complexes stabilize and facilitate the cortical transfer of newly encoded memories. More recently, Bollmann and colleagues [66] advanced this concept by showing that during prolonged rest periods (17–20 h) NREM sleep promotes the gradual drift of neuronal ensembles in the hippocampus towards representations of novel recall patterns. Neuronal ensembles drift as a consequence of a dynamic refinement of synaptic strength, where certain subsets become weakened while others strengthened—specifically during SWRs—resulting in a progressively refined ensemble composition. The role of Arc within this integrative framework is of significant interest, as it is proposed to function as an inverse synaptic tag that selectively weakens inactive synapses during post-learning consolidation periods [22]. This process enables the refinement of synaptic strengths through a balance between potentiation and depotentiation. By the disruption of Arc–TARPγ2 interactions, RIPSYR could affect the ability of Arc to regulate synaptic AMPAR trafficking, impairing its inverse tagging capacity resulting in the dysregulation of the balance of potentiation and depotentiation necessary for optimal synaptic remodeling during SWR-associated consolidation phases. Accordingly, this molecular interference could result in incorrect ensemble stabilization and diminished representational drift during NREM sleep, which might underlie the diminished memory retrieval observed in our study. Future research employing electrophysiological recordings during post-learning sleep following the administration of RIPSYR will be necessary to further elucidate its proposed interactions and to more thoroughly examine the synapse-specific molecular actions in relation to its effects on systems-level memory consolidation. In rats that performed cheeseboard maze tests, medium dose RIPSYR treatment did not alter Arc level significantly in CA1, CA3, as we found in the case of LTP as well, but it was elevated in the CC, which is known to play an important role in remote spatial memory retrieval [38]. There was no increase in the cerebellum, used as a control area, as it is not involved in spatial learning. However, the impact of RIPSYR on CA1 and CA3 cannot be ruled out, due to the high SD and an increasing trend of Arc levels measured by Western blot.

In vivo application of a small molecule may reveal previously unknown binding sites that are not revealed in in vitro systems. The probability of non-specific binding to other targets depends on the length of the peptide. Based on the fact that RIPSYR is a highly charged hexapeptide, it may bind several synaptic proteins as revealed in our immunoprecipitation study. We emphasize that the set of proteins identified here are candidates for interaction with the RIPSYR peptide under the experimental conditions we used. Without additional validation, these should not be interpreted as confirmed physiological partners of the peptide. Proteins with the highest abundance ratio identified in the immunoprecipitation study were regulators of actin and they are involved in neurite outgrowth and cell shape regulation. These include Basp1, which is implicated in synapse maturation and maintenance, and stimulates growth cone formation through actin remodeling [67], Marcks protein, which is involved in plasticity and learning [68], and Cdc42, which is a Rho GTPase that has an important role in dendritic spine plasticity [69,70]. Although we initially focused on Arc–TARPγ2 disruption, the presence of these regulators of plasticity indicates that off target interactions could contribute to the LTP and memory phenotype we observe. RIPSYR also bound AMPAR subunit GluA2 and GABA receptor (GABAR) ion channel subunits, such as Gabra1, Gabra2, Gabrb2, Gabrb3, which raised the possibility that RIPSYR may competitively inhibit glutamate and GABA binding to their receptors. These receptors directly govern synaptic strength and could independently alter LTP and memory consolidation, further raising the possibility that off-target interactions may contribute to our in vivo results. However, our in silico modelling of RIPSYR binding to AMPAR and GABAR subunits suggests that the binding sites of RIPSYR on these proteins are not at their endogenous ligand binding sites. In addition, the enthalpy changes upon RIPSYR binding to GluA2 are more favorable than Gabra1 based on the in silico results. Concerning the other binding partners of RIPSYR, the Basp1 and Cdc42 are important in neurite outgrowth and shaping the synapse [67,69]; thus, their interaction with RIPSYR might also contribute to declining memory consolidation. Notably, the inhibition of Cdc42 prevents the maintenance of LTP, but has no effect on LTP induction [71]. This is a limitation of using RIPSYR in vivo because effects of different additional binding proteins cannot be ruled out without conducting further studies.

## 5. Conclusions

In conclusion, we revealed that TARPγ2-derived RIPSYR peptide has physiological and behavioral effects in rats. Depending on its applied concentration, enhancement of potentiation in LTP and depleted memory retrieval were observed. These results indicate that RIPSYR has a distinct physiological effect, however, the exact molecular mechanism should be further investigated. While the observed in vivo effects on synaptic plasticity and spatial memory performance of RIPSYR indicate that the Arc-TARPγ2 binding and by the concomitant decrease in AMPAR mobility plays a functional role in these processes, one should be cautious when drawing mechanistic conclusions from these results alone. Although the initial rationale for the use of this peptide was the reported specificity to inhibit the Arc-TARPγ2 interaction, our immunoprecipitation and in silico study revealed additional binding partners of RIPSYR that could contribute to its in vivo effects. Therefore, we should not interpret the convergence of effects observed at the electrophysiological and behavioral levels as definitive evidence of a single mechanism. Instead, a complex outcome of altered signaling networks involving other proteins associated with the peptide may be reflected in these results. The extent of the contribution of Arc independent processes to the physiological and behavioral effects of RIPSYR needs further investigation. Moreover, Arc in complex with TARPγ2 cannot form capsids [26] indicating the overlap of TARPγ2 binding and capsid forming sites. Investigation of RIPSYR regarding Arc capsids in physiological processes has to be further elucidated in the future.

## Figures and Tables

**Table 1:** Docking scores (fastdfire algorithm) and RMSD values of the highest scored models.

Model ID	fastdfire Score	RMSD (Å)
s21ld149	13.02	2.825
s40ld191	13.25	16.439
s36ld24	13.23	16.854
s0ld75	13.40	17.241

## Data Availability

The raw data supporting the conclusions of this article will be made available by the authors on request.

## Supplementary Materials

The following supporting information can be downloaded at https://www.mdpi.com/article/10.3390/brainsci15080881/s1, Figure S1: Supplementary LTP results; Table S1: Synaptic proteins identified by LC-MS (mitochondrial, nuclear and contaminant-filtered).

## Abbreviations

The following abbreviations are used in this manuscript:

AMPAR	α-amino-3-hydroxy-5-methyl-4-isoxazolepropionic acid receptor
Arc	Activity-regulated cytoskeleton associated protein
TARP	Transmembrane AMPAR regulatory protein
LC-MS	Liquid chromatography–mass spectrometry
LTP	Long-term potentiation
LTD	Long-term depression
PSD95	Postsynaptic density protein 95
NMDAR	*N*-methyl-D-aspartate receptor
GluA2	AMPA-selective glutamate receptor 2
Gabra1	GABA receptor subunit alpha 1
HFS	High-frequency stimulation
PTP	Post-tetanic potentiation
eLTP	Early phase of LTP
fEPSP	Field excitatory postsynaptic potential
aCSF	Artificial cerebrospinal fluid
CC	Cingulate cortex
CRB	Cerebellum
LBD	Ligand binding domain
RMSD	Root mean square deviation
RMSF	Root mean square fluctuation
