# Supplementary material for: TARPγ2-Derived Peptide Enhances Early-Phase Long-Term Potentiation and Impairs Memory Retention in Male Rats
Source: Brain Sci. 2025 Aug 18;15(8):881. doi: 10.3390/brainsci15080881 (PMC12385072; doi:10.3390/brainsci15080881)

# Original western blot membranes

Cerebellum (CRB), cingulate cortex (CC), CA3 and CA1 region of the hippocampus of control and RIPSYR-peptide treated rats (superscript denotes the individual animal number) after the cheeseboard maze task.

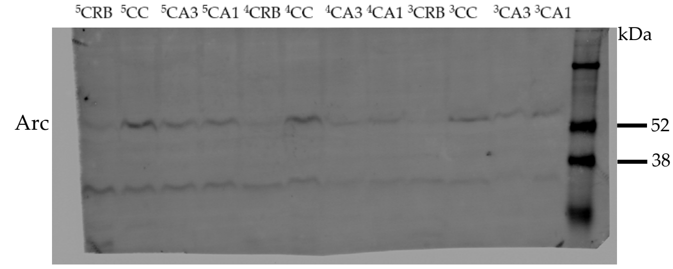


**Total protein content (Ponceau S stain)**

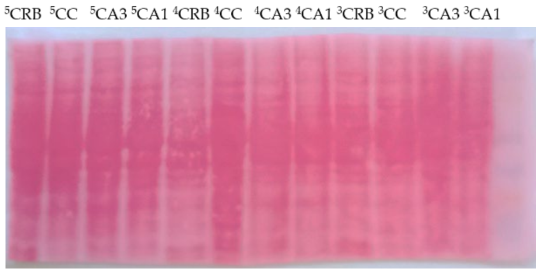


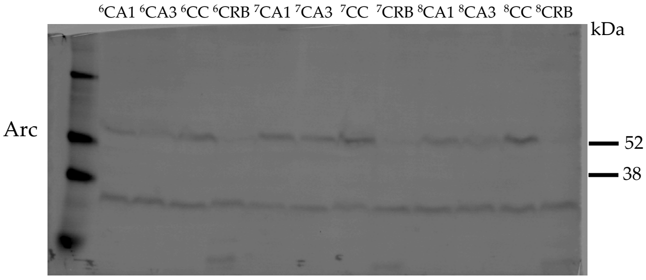


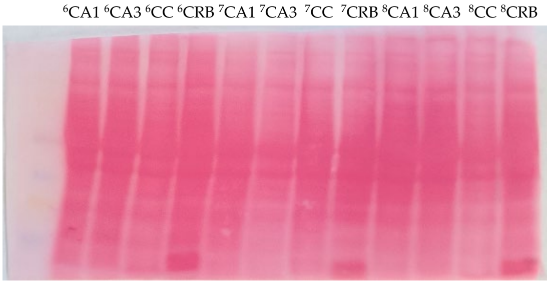


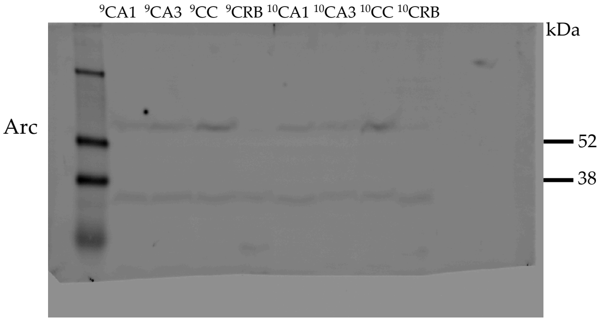


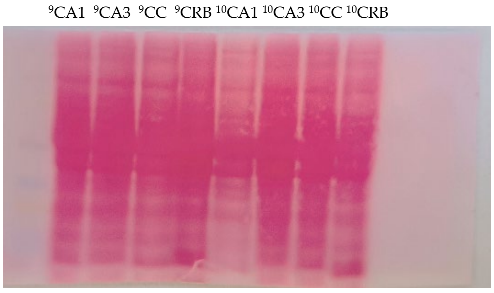


Western blot membranes of hippocampal samples from RIPSYR peptide–treated animals subjected to LTP induction.

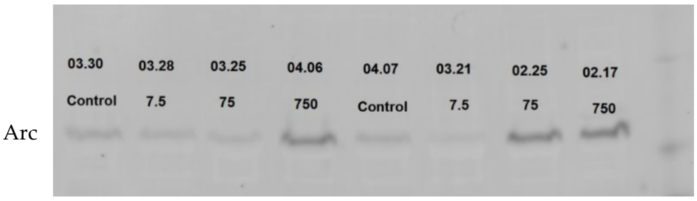


**Total protein content (Ponceau S stain)**

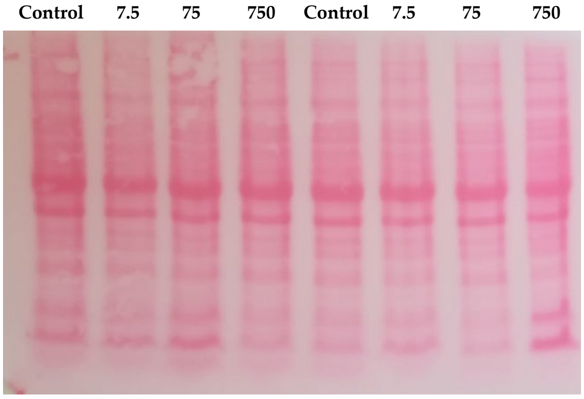

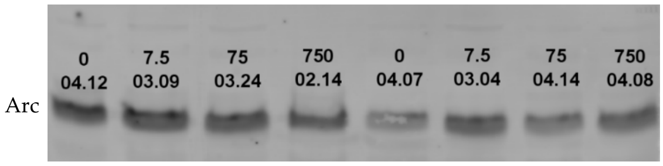


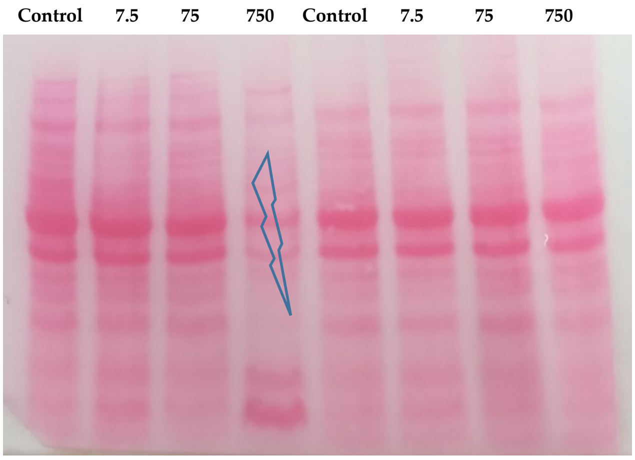


The sample was homogenized with a different buffer, we excluded this from the calculation.

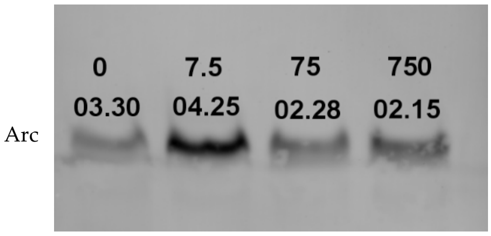


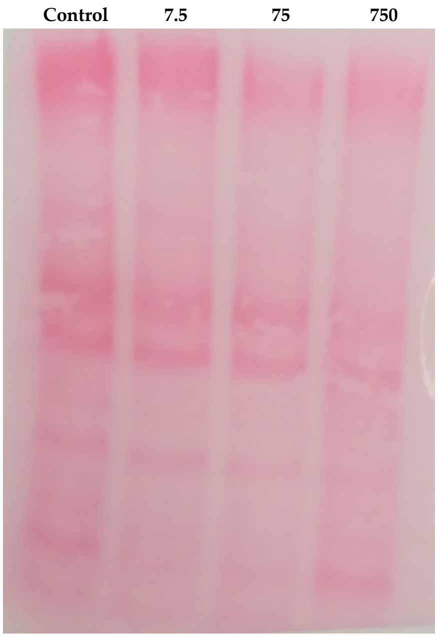

Supplement: Supplementary file 1 [file brainsci-15-00881-s001.zip › Original_blots_Mátyás_et_al_REVISED.pdf]
