# Supplementary material for: TARPγ2-Derived Peptide Enhances Early-Phase Long-Term Potentiation and Impairs Memory Retention in Male Rats
Source: Brain Sci. 2025 Aug 18;15(8):881. doi: 10.3390/brainsci15080881 (PMC12385072; doi:10.3390/brainsci15080881)
Supplement: Supplementary file 1 [file brainsci-15-00881-s001.zip › Supplementary_Mátyás_et_al_REVISED.pdf]

Supplementary information

TARPγ2 -derived peptide enhances early synaptic potentiation but impairs memory retention in rats

Dominik Mátyás [1,2,3], Vanda Tukacs [1,3], Vilmos Tóth [4], Péter Baracskay [1], Stefánia Krisztina Pap [1,2], Pál Stráner [5,6], Trần Minh Hiền [5,6], Éva Hunyadi-Gulyás [7], Zsuzsanna Darula [7,8], András Perczel [5,6], Katalin Adrienna Kékesi [1,2,3], Gábor Juhász [1,3]

[1]Laboratory of Proteomics, Institute of Biology, ELTE Eötvös Loránd University, Pázmány Péter sétány 1/C, Budapest, 1117, Hungary
[2]Department of Physiology and Neurobiology, Institute of Biology, ELTE Eötvös Loránd University, Pázmány Péter Sétány 1/C, Budapest, 1117, Hungary.
[3]InnoScience Hungary Ltd., Bátori út 9, H-3142 Mátranovák, Hungary
[4]ELTE NAP Neuroimmunology Research Group, Department of Biochemistry, Institute of Biology, ELTE Eötvös Loránd University, Budapest, Hungary
[5]Laboratory of Structural Chemistry and Biology, Institute of Chemistry, ELTE Eötvös Loránd University, Pázmány Péter sétány 1/A, H-1117 Budapest, Hungary
[6]HUN-REN–ELTE Protein Modeling Research Group, ELTE Eötvös Loránd University, Pázmány Péter sétány 1/A, H-1117 Budapest, Hungary
[7]Core Facility Proteomics Research Group, HUN-REN Biological Research Centre, Temesvári körút 62. H-6726 Szeged, Hungary
[8]Single Cell Omics Advanced Core Facility, Hungarian Centre of Excellence for Molecular Medicine, Temesvári körút 62. H-6726 Szeged, Hungary

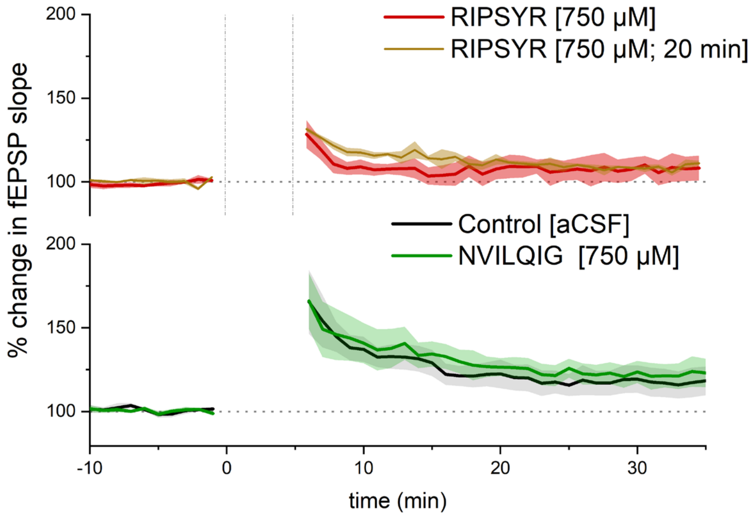

Figure S1. Supplementary LTP results. Control experiments with (upper panel) delayed HFS after high dose RIPSYR administration showed similar reduction in potentiation and (lower panel) with control peptide administration in high concentration showed no notable difference in LTP.

Table S1: Synaptic proteins identified by LC-MS (mitochondrial, nuclear and contaminant-filtered)

**Accession**	**Description**	**Abundance Ratio: (AP) / (CTRL)**	**Abundance Ratio Variability [%]: (AP) / (CTRL)**
A0A0G2K1L8	Brain acid soluble protein 1 OS=Rattus norvegicus OX=10116 GN=Basp1 PE=1 SV=1	**2.588**	18.69

Q05175	Brain acid soluble protein 1 OS=Rattus norvegicus OX=10116 GN=Basp1 PE=1 SV=2	**2.274**	18.39
F1LMW7	Myristoylated alanine-rich C-kinase substrate OS=Rattus norvegicus OX=10116 GN=Marcks PE=1 SV=3	**1.815**	47.84
P02688	Myelin basic protein OS=Rattus norvegicus OX=10116 GN=Mbp PE=1 SV=3	**1.789**	63.62
M0R6L8	DnaJ heat shock protein family (Hsp40) member C19 OS=Rattus norvegicus OX=10116 GN=Dnajc19 PE=1 SV=1	**1.744**	19.88
Q8CFN2	Cell division control protein 42 homolog OS=Rattus norvegicus OX=10116 GN=Cdc42 PE=1 SV=2	**1.706**	49.1
Q63666	Vesicle-associated membrane protein 1 OS=Rattus norvegicus OX=10116 GN=Vamp1 PE=1 SV=2	**1.656**	20.68
Q6JAM9	Transmembrane protein 35A OS=Rattus norvegicus OX=10116 GN=Tmem35a PE=1 SV=1	**1.562**	45.02
Q9WUW2	Synaptobrevin-2 OS=Rattus norvegicus OX=10116 GN=vamp2 PE=2 SV=1	**1.559**	23.45
Q0VGJ9	Receptor expression-enhancing protein OS=Rattus norvegicus OX=10116 GN=Reep2 PE=2 SV=1	**1.551**	35.24
I7EFB0	Myelin basic protein (Fragment) OS=Rattus norvegicus OX=10116 GN=Mbp PE=2 SV=1	**1.547**	42.71
F1LSY2	Neuronal pentraxin receptor OS=Rattus norvegicus OX=10116 GN=Nptxr PE=1 SV=1	**1.507**	25.8
P20171	GTPase HRas OS=Rattus norvegicus OX=10116 GN=Hras PE=1 SV=2	**1.505**	26.42
F1LMU7	GABA(A) receptor subunit alpha-2 OS=Rattus norvegicus OX=10116 GN=Gabra2 PE=3 SV=3	**1.503**	31.69
Q63754	Beta-synuclein OS=Rattus norvegicus OX=10116 GN=Sncb PE=1 SV=1	**1.491**	65.44
P08082	Clathrin light chain B OS=Rattus norvegicus OX=10116 GN=Cltb PE=1 SV=1	**1.429**	16.57
P62813	Gamma-aminobutyric acid receptor subunit alpha-1 OS=Rattus norvegicus OX=10116 GN=Gabra1 PE=1 SV=1	**1.397**	38.45
Q9QZM5	Abl interactor 1 OS=Rattus norvegicus OX=10116 GN=Abi1 PE=1 SV=3	**1.367**	26.32
P82471	Guanine nucleotide-binding protein G(q) subunit alpha OS=Rattus norvegicus OX=10116 GN=Gnaq PE=2 SV=2	**1.365**	22.25
P47971	Neuronal pentraxin-1 OS=Rattus norvegicus OX=10116 GN=Nptx1 PE=1 SV=1	**1.365**	9.79
Q45QL6	Guanine nucleotide binding protein beta 2 (Fragment) OS=Rattus norvegicus OX=10116 GN=Gnb2 PE=2 SV=1	**1.364**	30.26
P63138	Gamma-aminobutyric acid receptor subunit beta-2 OS=Rattus norvegicus OX=10116 GN=Gabrb2 PE=1 SV=1	**1.344**	44.41

P63322	Ras-related protein Ral-A OS=Rattus norvegicus OX=10116 GN=Rala PE=1 SV=1	**1.338**	30
G3V914	Glutamate receptor 2 OS=Rattus norvegicus OX=10116 GN=Gria2 PE=4 SV=2	**1.328**	33.67
Q05759	cAMP-dependent protein kinase catalytic subunit beta (Fragment) OS=Rattus norvegicus OX=10116 PE=2 SV=1	**1.32**	21.94
A0A0G2K0J9	Gamma-aminobutyric acid receptor subunit beta-3 OS=Rattus norvegicus OX=10116 GN=Gabrb3 PE=1 SV=1	**1.316**	47.91
D3ZXH7	Aly/REF export factor OS=Rattus norvegicus OX=10116 GN=Alyref PE=1 SV=1	**1.301**	27.65
